# Inhibition of the Metabolic Degradation of Filtered Albumin Is a Major Determinant of Albuminuria

**DOI:** 10.1371/journal.pone.0127853

**Published:** 2015-05-26

**Authors:** Julijana Vuchkova, Wayne D. Comper

**Affiliations:** 1 Department of Biochemistry and Molecular Biology, Monash University, Clayton, Victoria 3800, Australia; 2 SalAqua Diagnostics Inc., Suite 258, 888c 8^th^ Ave, New York, New York, 10019, United States of America; University of Utah School of Medicine, UNITED STATES

## Abstract

Inhibition of the degradation of filtered albumin has been proposed as a widespread, benign form of albuminuria. There have however been recent reports that radiolabeled albumin fragments in urine are not exclusively generated by the kidney and that in albuminuric states albumin fragment excretion is not inhibited. In order to resolve this controversy we have examined the fate of various radiolabeled low molecular weight protein degradation products (LMWDPs) introduced into the circulation in rats. The influence of puromycin aminonucleoside nephrosis on the processing and excretion of LMWDPs is also examined. The status and destinies of radiolabeled LMWDPs in the circulation are complex. A major finding is that LMWDPs are rapidly eliminated from the circulation (>97% in 2 h) but only small quantities (<4%) are excreted in urine. Small (<4%) but significant amounts of LMWDPs may have prolonged elimination (>24 h) due to binding to high molecular weight components in the circulation. If LMWDPs of albumin seen in the urine are produced by extra renal degradation it would require the degradation to far exceed the known catabolic rate of albumin. Alternatively, if an estimate of the role of extra renal degradation is made from the limit of detection of LMWDPs in plasma, then extra renal degradation would only contribute <1% of the total excretion of LMWDPs of albumin. We confirm that the degradation process for albumin is specifically associated with filtered albumin and this is inhibited in albuminuric states. This inhibition is also the primary determinant of the massive change in intact albuminuria in nephrotic states.

## Introduction

Albuminuria is a ubiquitous phenomenon associated renal disease. Its mechanism is still subject to elucidation. There is the traditional view that it is primarily the result of changes in glomerular permeability [1] whereas others have recently proposed that it is due, in part, to dysfunction in the proximal tubule retrieval pathway for filtered albumin [2,3,4]; both these mechanisms have serious ramifications as they both will potentially lead to hypoalbuminemia.

A benign form of albuminuria, that does not lead to hypoalbuminemia, has been proposed as a result of inhibition of kidney specific degradation of filtered albumin [5,6,7]. There is support for this mechanism as both proximal tubule cells [8,9,10] and podocytes [11,12] have the ability to endocytose albumin and degrade it through lysosomal activity [10,11,13]. It has been known for some time that albumin fragments are excreted in urine *in vivo* [5,14,15,16,17,18] and in urine from the isolated perfused kidney [13,19]; the capacity of this pathway corresponds to a fractional clearance of albumin prior to degradation of ~0.001 in rats [6]. In albuminuric states albumin fragment excretion is inhibited [5,6,20,21,22,23]. In fact there is a highly correlated relationship between increasing levels of albuminuria in diabetic patients and decreasing levels of albumin fragment excretion [21]. There has been conflicting data however as to the source of urinary fragments. Extensive studies using highly sensitive detection measurements have failed to detect radiolabeled albumin fragments in the circulation for both filtering [5] and non-filtering kidneys [6]. On the other hand there are reports of albumin fragments being detected in the circulation [24–27] and that these are responsible for their appearance in urine [18]. Furthermore their appearance in urine is not inhibited in albuminuric states [18]. These conclusions were generated by observing the processing of intravenously injected [^125^I]-labeled albumin in mice [18].

In order to determine, without ambiguity, whether the inhibition is associated with specific renal degradation it is necessary to examine the fate of radiolabeled low molecular weight degradation products (LMWDPs) that may occur in plasma. Various models of LMWDPs are examined in this study including i) the intravenous injection of tryptic digests of radiolabeled albumin which have a similar size distribution to albumin fragments identified in urine [17] and ii) the metabolic products of circulating radiolabeled low molecular weight proteins (LMWPs) (lysozyme and cytochrome c are studied) that are known to be degraded in the kidney to peptides and amino acids that are returned to the circulation [28–33]. The major questions to be addressed here are i) is the residence of LMWDPs in plasma too short, due to rapid kidney filtration, to detect? ii) what is the proportion of the initial dose appears as LMWDPs in urine? and iii) does the presence of LMWDPs in plasma account for their appearance in urine? In order to address whether albumin fragment excretion is inhibited in nephrotic states we also examine their processing in an experimental nephrotic podocytopathological condition induced by puromycin aminonucleoside (PA) that generates oxygen radicals [34] known to cause loss of organization of the interdigitating foot processes of the podocyte and podocyte detachment from the glomerular basement membrane [35].

## Materials and Methods

### Experimental animals

All animal experiments were approved by the Monash University Animal Ethics Committee, and conducted in accordance with their guidelines. Male Sprague-Dawley rats, 350–450 g in weight (10–12 weeks in age), were obtained from the Monash University Central Animal House (Melbourne, Australia). Throughout the experimental period, the rats were maintained under a 12-h day/night cycle with free access to standard rat food and water.

### Induction of puromycin aminonucleoside nephrosis

Induction of puromycin aminonucleoside nephrosis (PAN) was performed as previously described [20]. Rats were injected intravenously, via the tail vein, with puromycin aminonucleoside (PA; Sigma, St Louis, MO) (as a 3.5% solution in phosphate-buffered saline (PBS)) at a concentration of 10 mg/100 g body weight [20]. Rats were housed in rat boxes under a 12-h day/night cycle. Urine was collected in metabolic cages over a 24-h period at baseline (day 0) and days 5 and 8 following PA administration. Total urinary protein excretion was determined (Biuret assay [36]) at each of these days to ensure the onset of proteinuria. Those rats that were excreting more than 400 mg/24 h by day 8 were considered proteinuric and were included in the study. Peak proteinuria was generally achieved 9 days following PA administration. Experiments were therefore performed on day 9.

### Radioisotopes

Sodium boro-[^3^H]hydride (11.7 Ci/mmol), [^14^C]-formaldehyde (54 mCi/mmol) and [^14^C]inulin (2.4 mCi/g) were obtained from NEN Research Products, Du Pont, Wilmington, Delaware, USA. Tritium labeled water (0.25 mCi/g) was obtained from NEN Life Science Products, Boston, Massachusetts, USA.

### Tritium labeling of proteins

The proteins lysozyme (from chicken egg white; MW = 14,300 Da; Sigma, St. Louis, MO) and rat serum albumin (RSA) (MW = 69,000 Da; Sigma, St Louis, MO) were labeled with tritium (sodium boro-[^3^H]hydride; NEN, Du Pont, Wilmington, Delaware) using the reductive methylation procedure described by Tack *et al* [37]), with the reagents optimised for each protein. The specific activity of each labeled preparation is shown in Table 1. The structural integrity of the labeled proteins was checked by size exclusion chromatography.

**Table 1:** Specific activities of radiolabeled preparations, expressed as Becquerels per milligram of sample (Bq/mg).

Molecule	Specific Activity (Bq/mg)
[^3^H]lysozyme	4.8 × 10^6^
[^14^C]cytochrome c	1.2 × 10^5^
[^14^C]albumin	1.1–1.3 × 10^5^
[^3^H]albumin	1.2 × 10^6^
[^3^H]albumin peptides	1.2 × 10^6^

### Carbon-14 labeling of proteins

RSA and cytochrome c (type IIA from horse heart; MW = 12,400 Da; Sigma, St. Louis, MO) were labeled with carbon-14 ([^14^C]-formaldehyde; NEN, Du Pont, Wilmington, Delaware) using the reductive methylation procedure described by Eng [38]. The specific activity of each labeled preparation (Table 1) was determined by analysis of the radioactivity in the solution and analysis of the protein content, as determined by the Biuret assay [36]. The structural integrity of the labeled proteins was checked by size exclusion chromatography. Previous studies have demonstrated that [^14^C]-labeled albumin was processed by the kidney in essentially the same way as endogenous albumin [6].

### Trypsin digestion of [^3^H]-albumin

Digestion of albumin was performed using trypsin at a ratio of 6 mg trypsin/g of albumin as described by Strong *et al* [17]. The solution was then filtered through a 30,000 Da molecular weight cut-off membrane. The major labeled components of the tryptic digest had molecular weights <700 Da [17].

### Processing of proteins *in vivo*–short term study using bolus intravenous dose

Normal and experimentally induced nephrotic male Sprague-Dawley rats were injected with each particular radiolabeled tracer by a bolus injection into the tail vein. Blood pressure was monitored. For the subsequent duration of the experimental period, rats were maintained in individual metabolic cages. Urine was collected over the duration of the experimental period. Blood samples were taken from the tail vein at various times post injection. At the end of the experimental period, the rats were anaesthetised with an intraperitoneal injection of pentobarbitone sodium (20 mg) and sacrificed by cardiac puncture. The remaining urine in the bladder was collected. Selected organ tissues (kidneys, spleen, liver, muscle) were excised from the body, digested and analyzed for radioactivity to determine the overall distribution and possible renal tissue accumulation of each labeled tracer.

*In vivo* administration of the radiolabeled tracer into the circulation of normal rats by bolus intravenous injection were as follows; [^14^C]RSA 3.3x10^5^ Bq, 2.53 mg; [^3^H]RSA 1.6x10^6^ Bq 1.36 mg; [^14^C]cytochrome c 3.3x10^4^ dpm, 0.29 mg; [^3^H]lysozyme 1.6x10^6^ Bq, 0.33 mg; [^3^H]RSA peptides 1.6x10^6^ Bq, 1.36 mg. The following time points (h) of 0.25, 0.5, 1, 2, 4, and 24 were employed as the length of time for which the radiolabeled tracer was in the circulation before sacrificing the animal.

### Preparation of plasma and urine for analysis

All urine and blood samples collected were centrifuged at 3500 rpm for 15 min to obtain sediment-free urine and plasma, respectively. The urine and plasma samples were then analyzed for radioactivity and total protein content (Biuret assay [36]). The integrity of the radioactive material in each sample was analyzed by size exclusion chromatography.

### Digestion of rat organs

At the end of the experimental period (following cardiac puncture), organ tissues such as the kidney, liver, spleen and muscle were excised from the rats, washed externally with PBS and weighed. Each tissue (whole kidney, whole spleen, 1–2 g liver, 1–2 g muscle) was minced and 1.4 M NaOH was added to make a final volume of 6 ml. The samples were covered loosely and immersed in boiling water for 15–30 min, to allow complete digestion of the organ tissues. Three 100 μl sample aliquots of each organ digest were taken, to which was added 50 μl of hydrogen peroxide (30% w/v), to decolourise the samples. The volume of each sample was made up to 1 ml with distilled water before adding scintillation fluid. Before counting for radioactivity, the samples were stored in the dark overnight to minimise chemiluminescence interference with radioactive counting. The presence of the radiolabeled tracer in the organ tissues was determined and expressed as disintegrations per minute per gram (dpm/g) of tissue, or as percentage of injected dose. When values are expressed as percentage of injected dose, it is assumed that total liver mass equals 2.5% of the body weight, and total muscle mass equals 25% of the body weight [33,39]. We made no attempt to comprehensively examine the distribution of radioactivity. Values expressed in Table 2 are similar to that obtained for the accumulation of radioactivity from radiolabeled albumin after 48 h as studied in rabbits [40].

**Table 2:** The percentage of administered radioactivity recovered in the plasma, urine, kidney and extra-renal (ER) tissues, at 2 h after injecting [^3^H]lysozyme, [^14^C]cytochrome c, [^14^C]RSA or [^3^H]RSA tryptic peptides.

	Plasma	Urine	Kidney	ER Tissue	Total
[^3^H]lysozyme (n = 5)	3.05±0.56	4.14± 2.54	35.10±4.99	12.36± 2.80	**54.65**±10.89
[^14^C]cytochrome (n = 5)	3.82±0.25	1.18± 0.69	5.86±2.30	18.28± 2.98	**29.14**±6.22
[^14^C]RSA (n = 5)	78.15±2.1	0.32± 0.15	1.85±0.25	7.29± 1.06	**87.61**±3.54
[^3^H]RSA tryptic peptides (n = 4)	4.05±0.24	1.53± 0.73	3.17±0.38	27.50± 0.88	**36.25**±2.23

The extra-renal tissues analyzed were muscle, spleen and liver. Values are mean (± SD).

### Size exclusion chromatography

Samples containing radiolabeled albumin were analyzed using a Sephadex G-100 (Pharmacia, Uppsala, Sweden) column (1.6 × 60 cm^2^), and those containing low molecular weight proteins were analyzed using a Sephadex G-50 (Pharmacia, Uppsala, Sweden) column (1.6 × 50 cm^2^). Samples were eluted with a PBS buffer (pH 7.4, containing 0.2% bovine serum albumin (BSA) (to minimise adsorption, and 0.02% azide to prevent bacterial contamination), at 4°C at a flow rate of 20 ml/h, and fractions of approximately 1.5 ml (Sephadex G-50) or 1.7 ml (Sephadex G-100) were collected, with recoveries greater than 90%. Each column was calibrated using blue dextran T2000 (weight average molecular weight ~ 2,000,000 Da), at a concentration of 2 mg/ml, and tritium water (NEN, Boston, Massachusetts) at a concentration of 4 × 10^4^ dpm/ml, to determine the void volume (V_o_) and total volume (V_t_) of the column, respectively. Samples for analysis were made up to 1 ml with elution buffer before applying to the top of the column. The available volume of material fractionated on the column (K_av_) was determined by the formula K_av_ = (V_e_-V_o_)/(V_t_—V_o_), where V_e_ is elution volume of material. Both columns were calibrated with standard molecules of known radii. For each column calibration, a linear relationship was obtained for the plot of radii versus K_av_ (r^2^ > 0.99). Other radii estimates were obtained by both interpolation and extrapolation of this plot.

### Determination of radioactivity

Radioactivity from [^3^H]-labeled or [^14^C]-labeled material was determined by beta scintillation counting in a Wallac 1409 liquid scintillation counter, using a 1:3 aqueous sample to Optiphase scintillant ratio. When samples contained both [^3^H]- and [^14^C]-labeled material, a dual label protocol was used to determine the radioactivity from each isotope. Each sample was counted for 1 min. The radioactivity was expressed as Becquerel (Bq), where correction was made for quenching, chemiluminescence and counter efficiency.

### Statistical analysis

All quantitative data are expressed as mean ± standard deviation (SD) unless stated otherwise, where n represents the number of determinations. Statistical differences were assessed using the two-tailed Student’s t-test for unpaired data, with a *P*-value of less than 0.05 denoting statistical significance. Linear regression analysis was performed using the computer program SigmaPlot (Version 4, for Windows 98, Jandel Corporation, San Rafael, California, USA) or Microsoft Excel.

## Results

In this section we will primarily feature the results obtained for lysozyme and albumin. Results for cytochrome c were substantially similar to those for lysozyme.

### Structural integrity of radiolabeled proteins in plasma and urine

**Fig 1:**
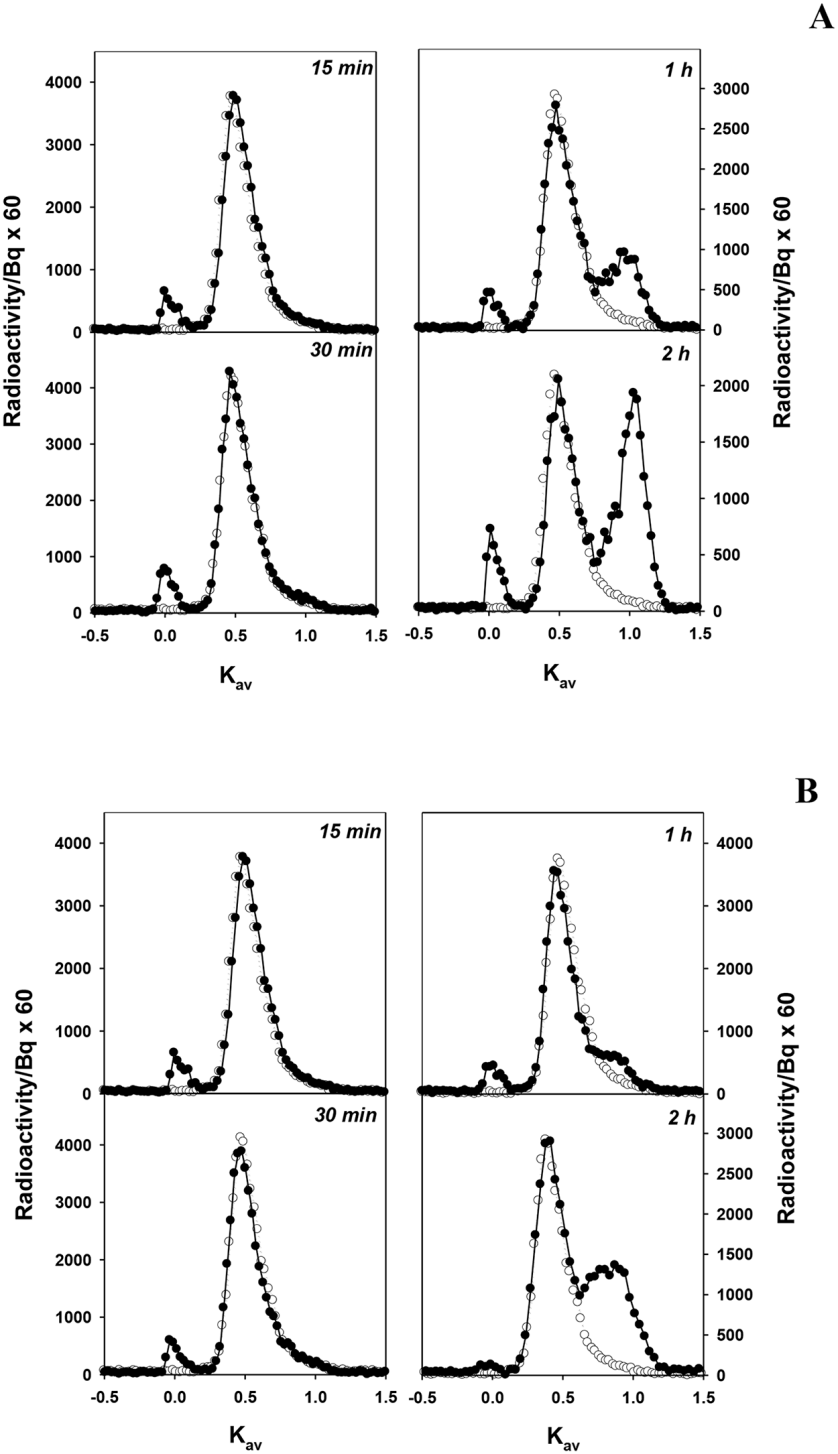
Representative size exclusion profiles of [^3^H]lysozyme in plasma (A) and in urine (B) taken at various times after intravenous administration in normal rats. The elution profile of native [^3^H]lysozyme (*open circles*) is shown for comparison. Chromatography was performed on Sephadex G-50. For both plasma and urine samples *n* = 3 for each time point.

After a bolus intravenous administration into the animals, the integrity of each circulating tracer was determined at various times thereafter by frequent sampling of plasma and subsequent analysis by size exclusion chromatography. Fig 1 shows the time development of the size exclusion profiles of radiolabeled material in plasma and urine over the 2 h period after intravenous administration of [^3^H]lysozyme. All the profiles demonstrate that some labeled material is eluting at the void volume (K_av_ = 0)(≥ 30,000 Da molecular weight) of the column in both plasma and urine samples, which is earlier than the normal elution of the LMWP. These results suggest that the labeled material was binding to high molecular weight material in the sample (observed also with cytochrome c). The potential binding of LMWPs to albumin is not the result of the use of the column elution buffer, which included 2 mg/ml albumin, as the purified LMWP eluted normally. However we cannot eliminate the binding of LMWDPs (ie., labeled peptides) to albumin in the elution buffer, as it also occurs in urine samples where the level of high molecular weight urinary proteins would be low.

Fig 1A demonstrates that intact, unbound material is also present as well as a significant quantity of LMWDPs material eluting at a Kav ~ 0.8–1.0 (< 1,000 Da molecular weight). With time, the amount of intact material decreased and the amount of degraded material increased. At 2 h ~40% of the radioactivity in plasma was associated with LMWDPs (Fig 1A). The appearance of the LMWDPs in the plasma over time is consistent with previous *ex vivo* studies, which have suggested that these products arise from degradation of the LMWP within the kidney and the release of those LMWDPs to the blood supply [28,29, 31,32].

The appearance of those LMWDPs in the urine was slow compared with their appearance in the plasma (Fig 1B). In fact, the ratio of intact protein to degradation products was always lower in the plasma than seen in the urine (Fig 1). This would mean that the plasma LMWDPs are not being exclusively filtered by the kidney but are being deposited in other regions of the body. This is likely to contribute to the fact that the total radioactivity administered to rats could not be completely accounted for (Table 2) and only small quantities could be recovered in urine.

**Fig 2:**
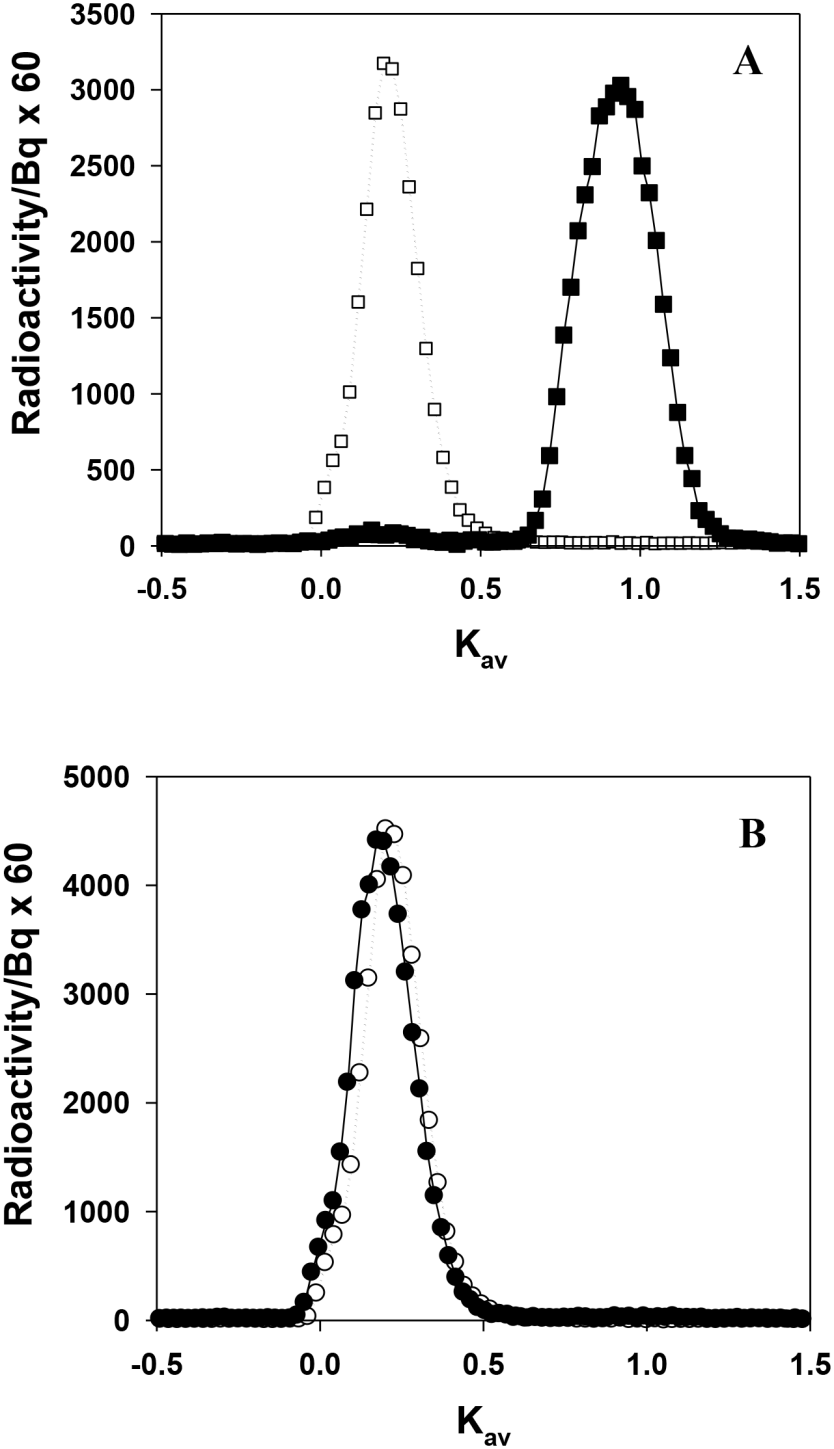
**(A)** Representative size exclusion profile of [^14^C]RSA in urine (*closed squares*). The profiles of urine collected at 0.5 h, 1 h and 2h were identical. For each time point *n* = 5. Dotted line represents native [^14^C]RSA (*open squares*). **(B)** Representative size exclusion profile of [^14^C]RSA in plasma (*closed circles*). Identical profiles were obtained at 15 min, 30 min, 1 h, 2 h, 24 h and 168 h after intravenous administration in rats. For each time point *n* = 5 to 7. Dotted line represents native [^14^C]RSA (*open circles*). Chromatography was performed on Sephadex G-100.

Chromatographic analysis of urine collected over the 2 h period after administration of [^14^C]RSA shows significant and relatively rapid (as compared to LMWPs) degradation of radiolabeled albumin to peptides, with <5% of albumin remaining intact (Fig 2A), which is consistent with previous studies [5,15,16,20,21]. Unlike LMWPs, there was no indication of labeled material eluting at the void volume (albeit with a higher exclusion limit >200,000 Da molecular weight). Studies of rats with non-filtering kidneys have previously confirmed that these albumin fragments are also produced specifically by the kidney and not by extra renal sources [6].

Again in contrast to LMWP, chromatographic analysis of plasma, taken at various times over a 168 h period after administration of [^14^C]RSA, shows that circulating albumin remained intact and not aggregated or degraded (Fig 2B). In fact, the area under the intact albumin peak was ~1,200 Bq which is significantly greater than the area under the fragment peak of <0.3 Bq. This is clearly contrary to the amount of [^125^I]-labeled low molecular weight material seen in plasma by Weyer et al [18] at a level of ~1% of the labeled material. It does confirm studies by Osicka and Comper [5] who analyzed plasma that contained 30 times more radiolabeled albumin than the Weyer et al [18] study, and no low molecular weight material could be detected (sensitivity down to at least 0.003%). This has been confirmed in human studies where albumin peptides can be found in urine [17,21] but not in plasma [21].

### Plasma elimination and tissue distribution

**Fig 3:**
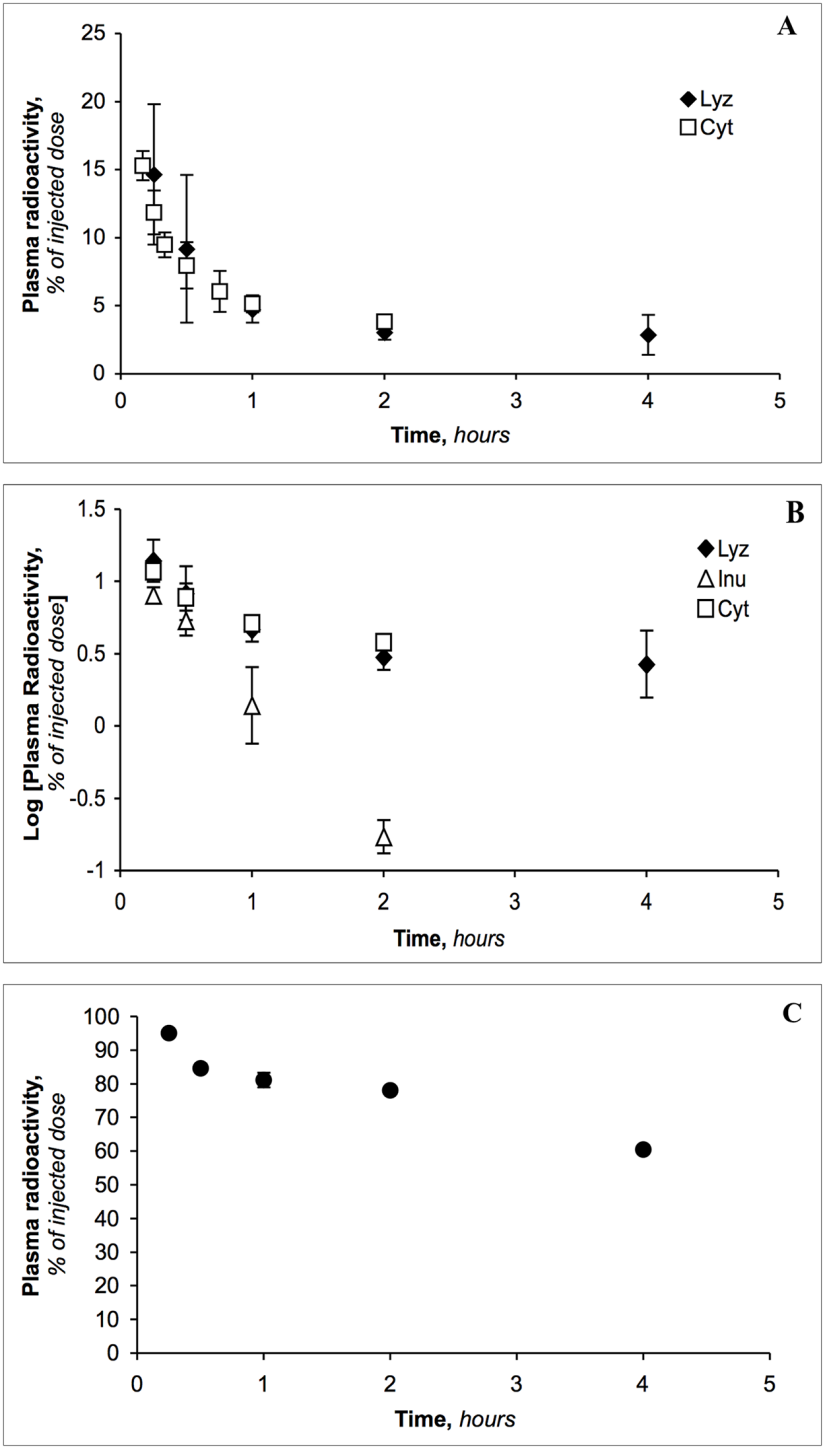
**(A)** Plasma radioactivity as a function of time after intravenous administration of [^3^H]lysozyme (*n* = 9, 10, 8, 6, and 2 at 0.25, 0.5, 1, 2 and 4 h, respectively) and [^14^C]cytochrome c (*n* = 4, 9, 4, 14, 4, 10 and 5 at 0.17, 0.25, 0.33, 0.5, 0.75, 1 and 2 h, respectively). **(B)** Log plasma radioactivity as a function of time after intravenous administration of [^3^H]lysozyme (*n* = 9, 10, 8, 6, and 2 at 0.25, 0.5, 1, 2 and 4 h, respectively), [^14^C]cytochrome c (*n* = 9, 14, 4, 10 and 5 at 0.25, 0.5, 1 and 2 h, respectively) and [^14^C]inulin (*n* = 7, 11, 7 and 5 at 0.25, 0.5, 1 and 2 h, respectively). **(C)** Plasma radioactivity as a function of time after intravenous administration of [^14^C]RSA (*n* = 5 at each time point). The percentage of injected dose remaining in the circulation was calculated by assuming that the total blood volume equals 7% of the body weight [33,39]; % injected dose = [(Bq/ml x total ml of blood)/total Bq injected] x 100.

Following intravenous administration, the labeled LMWPs were rapidly cleared from the circulation (Fig 3A) (>90% in 15 mins), whereas the clearance of albumin was relatively slow (Fig 3C). The results in Fig 3A are consistent with previous studies [29,33,39,41,42] which showed similar plasma clearance patterns for LMWP. The new feature of these results is the biphasic nature of the elimination of LMWPs (Fig 3B). The small but finite amount of material seen at 4 h is not rapidly eliminated but remains even at 24 h; for lysozyme it was 3.34% (*n* = 8). This is most likely due to the binding to high molecular weight material seen in Fig 1A. On the other hand, a small inert molecule such as inulin (molecular weight = 5,200 Da) is completely cleared from the circulation within 2 h (Fig 3B).

**Fig 4:**
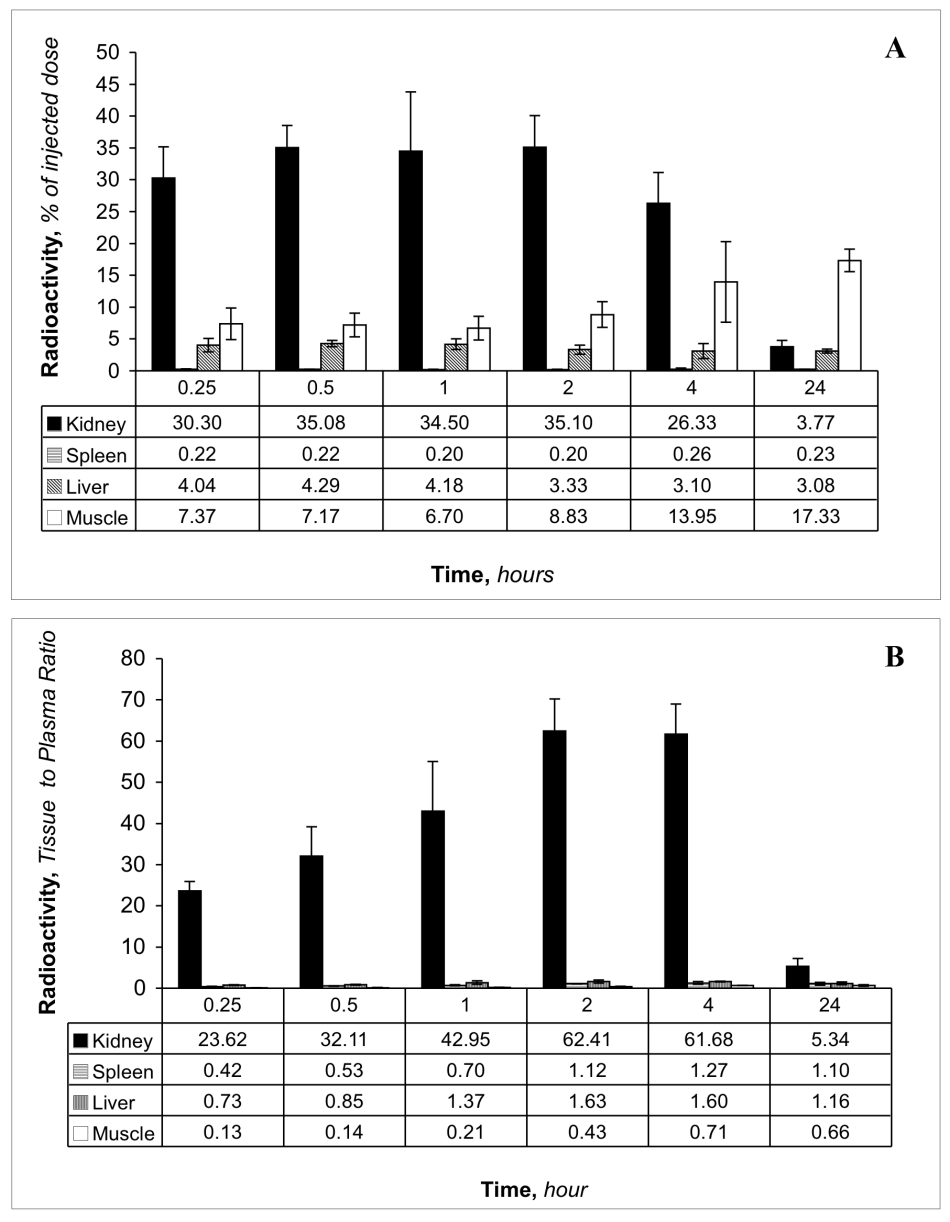
**(A)** Tissue distribution of radioactivity at various times after administration of [^3^H]lysozyme. Values are expressed as percentage of the injected dose, assuming total blood volume equals 7% of the body weight, total muscle mass equals 25% of the body weight and total liver mass equals 2.5% of body weight. **(B)** Tissue to plasma ratio of radioactivity at various times after intravenous administration of [^3^H]lysozyme. Tissue to plasma ratio was calculated as [(Bq/g tissue)/(Bq/ml plasma)]. For all tissues, *n* = 4, 5, 6, 6, 2 and 4 at 0.25, 0.5, 1, 2, 4 and 24 h, respectively.

The rapid loss of LMWP from the circulation can be accounted for, in part, by the rapid uptake by the kidney (Fig 4). Analysis of specific tissue distribution of radioactivity shows that the kidneys were the only organs to accumulate the LMWP substantially above plasma levels over the period of 15 minutes to 2 h (Fig 4B). The mean maximum kidney tissue to plasma ratios of radioactivity after administration of lysozyme and cytochrome c were 62.4 and 21.6, respectively. At 2 h we could account for 29–53% of the LMWPs in plasma, urine, kidneys and extra-renal tissues (Table 2).

**Fig 5:**
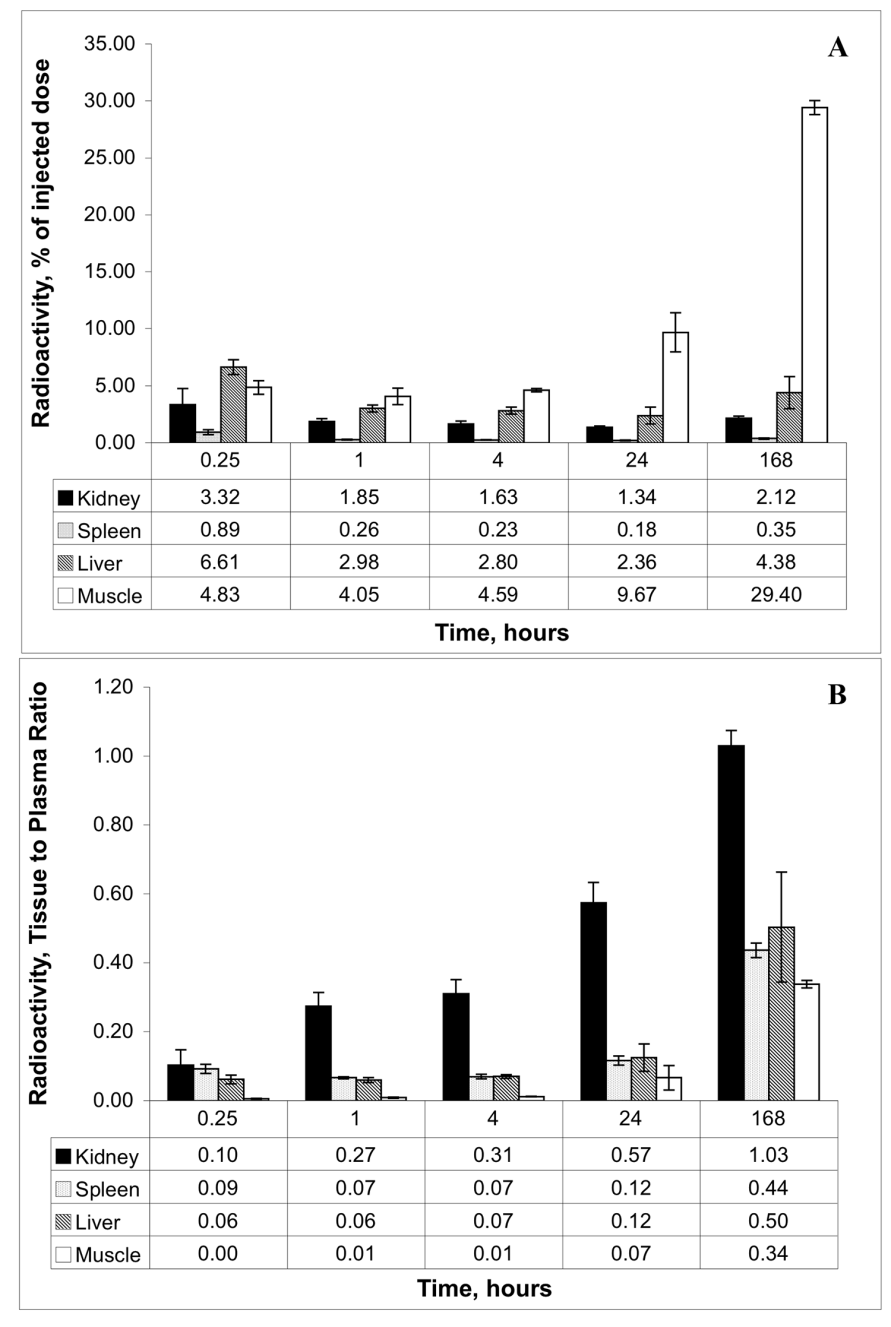
**(A)** Tissue distribution of radioactivity at various times after administration of [^14^C]RSA (at 1, 4, 24 and 168 h) or [^3^H]RSA (at 0.25 h). **(B)** Tissue to plasma ratio of radioactivity at various times after intravenous administration of [^14^C]RSA or [^3^H]RSA. Tissue to plasma ratio was calculated as [(Bq/g tissue)/(Bq/ml plasma)]. For all tissues **(A & B)**, *n* = 5 at each time point. Values are expressed as percentage of the injected dose, assuming total blood volume equals 7% of the body weight, total muscle mass equals 25% of the body weight and total liver mass equals 2.5% of body weight. Tissue to plasma ratio was calculated as [(dpm/g tissue)/(dpm/ml plasma)].

In contrast to the behaviour of LMWP, Fig 5A shows that there is essentially no accumulation of radioactivity in the kidney tissue after administration of labeled RSA, nor was there after 7 days although for the amount of radioactivity present in plasma, the kidney to plasma ratio (Fig 5B) did increase over time.

**Fig 6:**
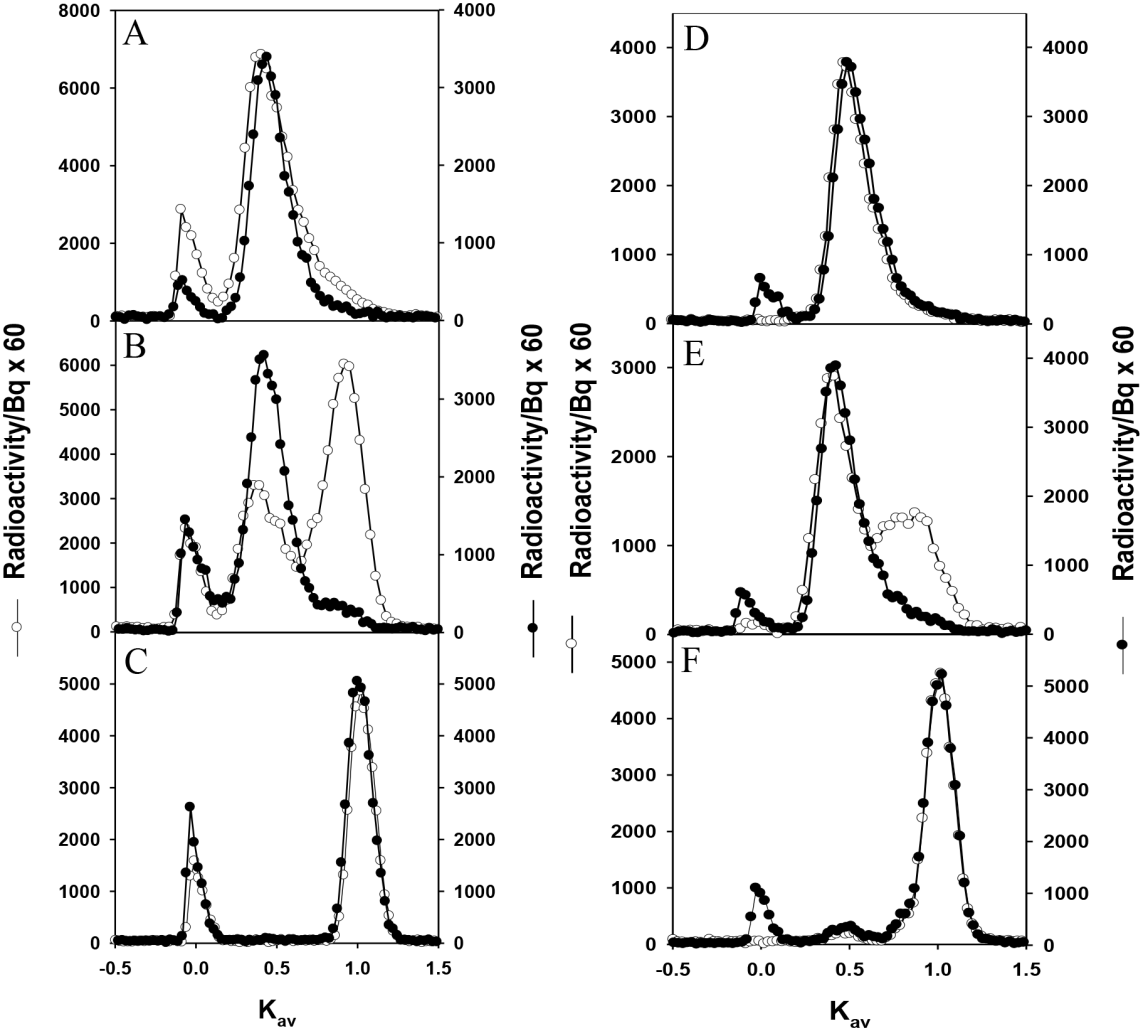
Representative size exclusion profiles of [^3^H]lysozyme in plasma taken at 0.25 h (A), 2 h (B), and 24 h (C), after intravenous administration in PAN (*closed circles*) and control (*open circles*) rats. Representative size exclusion profiles of [^3^H]lysozyme in urine collected at 0.25 h (**D**), 2 h (**E**), and 24 h (**F**). Native [^3^H]lysozyme elutes at Kav 0.46. Chromatography was performed on Sephadex G-50. For both plasma and urine profiles, *n* = 3 for each time point.

The percentage of administered radioactivity recovered in plasma, urine, kidney and selected tissues after a 2 h period is shown in Table 2. This Table features some important results. LMWPs and LMWDPs, which would be freely permeable in the kidney (certainly as studied in isolated perfused kidney preparations [33]), are not primarily destined for urinary excretion. In fact the percent of injected dose appearing in the urine collected over a 2 h period was 1.2 for cytochrome c and 4.1 for lysozyme; these values were similar to that obtained for exogenous albumin tryptic peptides (Table 2). Yet in spite of the relatively rapid elimination of LMWPS and LMWDPs we always identified small but finite quantities in the plasma after 2 h (see also Fig 1A) and at 24 h (Fig 6); this compares to the plasma clearance of a small molecule like inulin that was complete within 2 h (Fig 3B). This demonstrates that if LMWDPs exist in plasma they simply don’t rapidly disappear undetected—a small quantity has a finite residence time that allows for their detection. This is the result of binding to high molecular weight proteins as seen in Fig 1.

### Processing of proteins in nephrotic states

**Fig 7:**
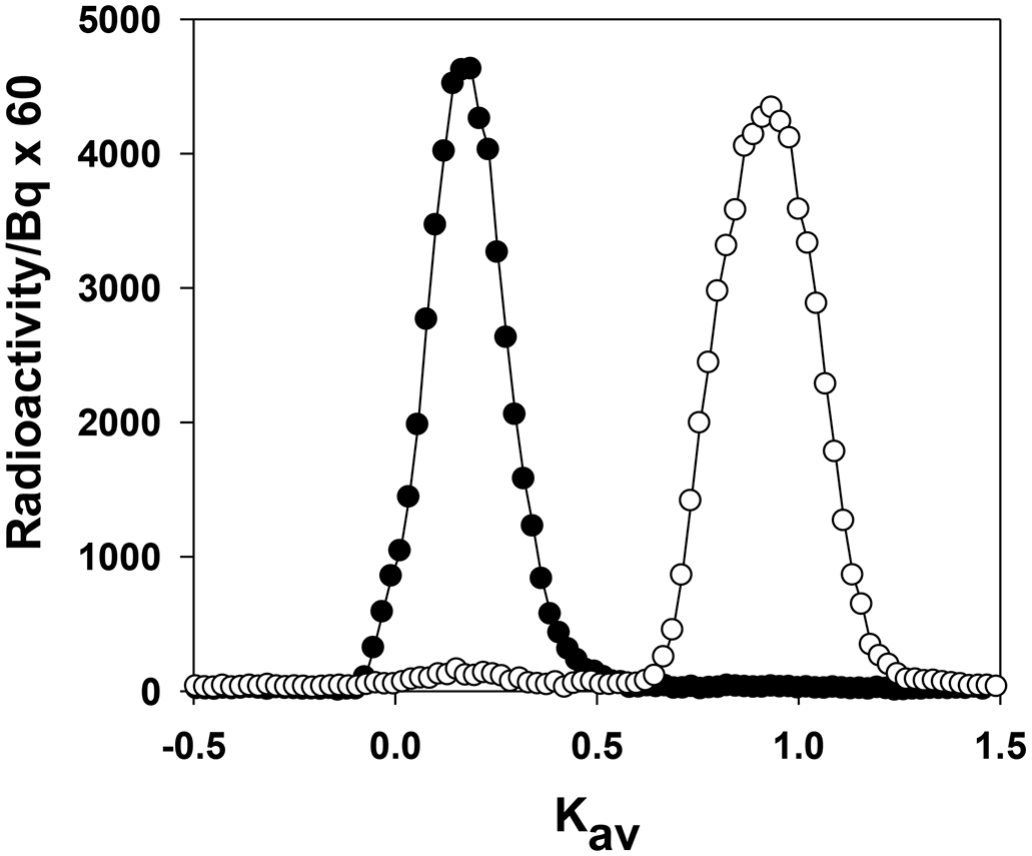
Representative size exclusion profiles of [^14^C]RSA in urine collected after intravenous administration in PAN (*closed circles*) and control (*open circles*) rats. The same levels of radioactivity in the urine of PAN and controls was loaded onto the column. Identical profiles were obtained at 2 h and 24 h (from *n* = 5 for each time point). Native [^14^C]RSA elutes at Kav 0.18. Chromatography was performed on Sephadex G-100.

The physiological parameters of the PA nephrosis (PAN) and control experimental groups are shown in Table 3. PAN increased the excretion of albumin but did not change the excretion of LMWP (Table 4), slowed but did not completely inhibit the appearance of LMWP degradation products in plasma and urine (Fig 6), whereas PAN completely inhibited any renal degradation of albumin (Fig 7). In fact, the area under the intact albumin peak in the PAN group, which was ~1,200 Bq which is significantly greater than the area under the fragment peak of <0.3 Bq.

**Table 3:** Physiological parameters of the PAN and control experimental groups.

	Total protein excretion/(mg/24h)	Urine flow rate/(ml/min)(10^-3^)
PAN		
[^3^H]lysozyme (*n* = 17)	627.9 ± 97.0^†^	15.8 ± 9.1*
[^14^C]RSA (*n* = 7)	732.0 ± 68.0^†^	13.2 ± 4.8
Control		
[^3^H]lysozyme (*n* = 14)	92.2 ± 16.6	8.3 ± 1.9
[^14^C]RSA (*n* = 7)	53.1 ± 9.6	8.8 ± 2.6

Total protein excretion was measured by the Biuret assay. Measurements were performed on rat urine collected on day 9 after intravenous administration of PA or PBS (for PAN and control groups, respectively).

^†^ = *P* < 0.001 and * = *P* < 0.005 versus control.

The rats were used to determine tissue distribution and excretion of material at different times.

**Table 4:** Urinary excretion of [^3^H]lysozyme and [^14^C]RSA in control and PAN rats, shown as Bq and total excretion in 24 h (as percentage of the injected dose of radioactivity).

	Urinary excretion	
	Bq	% of injected dose
PAN		
[^3^H]lysozyme (n = 5)	351 ± 94	15.20 ± 4.08
[^14^C]RSA (n = 7)	84 ± 16†	36.52 ± 6.97†
Control		
[^3^H]lysozyme (n = 5)	336 ± 52	11.18 ± 1.75
[^14^C]RSA (n = 7)	5 ± 1	3.50 ± 0.43

^†^ = *P* < 0.0001 versus control.

The appearance of material derived from LMWPs eluting at the void volume was apparent in all profiles (Fig 6). It is interesting that at 24 h when all the lysozyme had been converted to LMWDPs in both urine and plasma that material eluting at the void volume and the total volume still existed. It would be consistent with i) the binding of LMWDPs to high molecular weight material particularly in plasma and ii) the dissociation of the LMWDPs from the high molecular weight material on the column due to the law of mass action as the LMWDPs are normally cleared so quickly. The prolonged elimination of LMWDPs in PAN was similar to that in controls; the percentage of the initial dose of lysozyme in plasma at the times of 0.25 h, 2 h, 8 h, 16 h and 24 h was 18.2, 3.8, 2.85, 2.83 and 3.2 respectively (*n* = 12 for each time point). This result provides a cautionary note that simply identifying LMWDPs in the circulation does not mean that they will be rapidly eliminated or filtered by the kidney.

## Discussion

The most striking result of this study is that when LMWDPs are introduced into plasma and then cleared only low quantities (<4%) appear in urine (Table 2). The same result is obtained when either exogenous LMWDPs are specifically introduced into plasma or when LMWDPs are formed *in situ* and appear up to 4 h later (Fig 4) in plasma as a result of the metabolic degradation of exogenous LMWPs. The reason that LMWDPs, when present in non-steady state conditions as in [18], are not exclusively filtered by the kidney is that they rapidly equilibrate in various non-renal tissues; this compares with [^14^C]inulin that is preferentially filtered by the kidney (57.2 ± 12.6% (n = 4) appears in urine at 30 min). This is not surprising as small molecules are commonly used to measure whole body extracellular and intracellular volumes [43] and LMWDPs will distribute in the extracellular volume and to varying degrees in the intracellular volume. We also identify that in all cases a small proportion of LMWDPs may also be involved in binding to high molecular weight proteins in plasma. We do observe over time, once the plasma has been essentially cleared of LMWDPs, their level in urine may increase due to slow release from tissues; for lysozyme 11% of the injected dose appears after 24 h (Table 4).

In the Weyer et al [18] study the urinary excretion of radiolabeled LMWDPs from a bolus of intravenously administered [^125^I]-labeled albumin (1 x 10^6^ counts per minute (cpm)) was studied in normal mice and cubilin/megalin knockout mice. After 6 h, 77,000 cpm was detected in urine (~8% of the initial dose) for both the wild type and knockout mice as LMWDPs whereas the total amount of LMWDPs appearing in plasma after 6 h was ~3,200 cpm (0.3% of the initial dose) assuming a plasma volume of 1 ml [44]. In order to account for the appearance of LMWDPs in urine Weyer et al [18] assumed that LMWDPs were exclusively filtered by the kidney and that their appearance in urine could be accounted for by multiplying the time weighted average concentration of LMWDPs in plasma by the GFR. They assumed that the appearance of LMWDPs in plasma was a pseudo steady state value (ie., constantly being produced metabolically (~70,000 cpm in 6h) and removed by filtration (although there was no experimental evidence to support this)). It is clear that the assumption made by Weyer et al [18] would not apply to the behaviour of LMWDPs in rats. Specifically, we demonstrate that the destiny of LMWDPs identified in plasma may be directed towards either deposition in non-renal tissues or be involved in the binding to high molecular weight components in plasma or be directed towards kidney filtration.

We have previously performed an analysis of [^14^C]albumin processing in mice with the knowledge that it behaves like endogenous albumin [6]. We measured an albumin fragment excretion in mice over 24 h at 4.9 mg/24h [22]. Given that the plasma albumin is ~25% of the total albumin [44] this would mean that albumin fragment excretion would account for only 4% of the total body albumin processing in 24 h for a 20 g mouse. This compares favourably to the data in Table 4 for rats where we find that 3.5% of the injected dose of albumin appears in urine over 24h. In any case the excretion rate of LMWDPs in the mice calculated on the basis of radiolabeled material in the Weyer et al [18] study (comparison to endogenous albumin was not made) is high with the 8% of the initial dose found in urine after 6 h.

The excessive excretion of radiolabeled LMWDPs in urine in the Weyer et al [18] study raises a number of questions. Was the excessive excretion of LMWDPs due to deiodination of [^125^I]-labeled albumin in the kidney [45] and the excretion of free ^125^I? Another possibility is that [^125^I]-labeled albumin was denatured [46]. Denatured albumin is known to be rapidly cleared from the circulation [47]. Clavant et al [48] have demonstrated in the isolated perfused kidney that denatured albumin is degraded at ~20 times the rate of native albumin and these LMWDPs are excreted in urine. These renal centric processes, independent of megalin/cubilin, are likely to explain i) why there is excessive excretion of LMWDPs in the Weyer et al study [18] and ii) why a significant proportion of the LMWDPs in the Weyer et al [18] study appear in urine.

Weyer et al [18] make the argument that various reports have noted the presence of albumin fragments in plasma [24–27] and this is support for the notion that they are responsible for their appearance in urine. This is a not a strict representation as these fragments are relatively large (>5,000 Da [27] and > 20,000 Da [24–26]) as compared to the LMWDPs identified by Weyer et al [18] (<4,000 Da). Notably, Weyer et al did not identify any of these relatively large fragments. Furthermore, the 0.3% of radiolabeled LMWDPs seen in plasma in the Weyer et al study will only lead to a small increase in the excretion of LMWDPs, at least in rats, given that only 4% of the plasma material is excreted in urine. The appearance of LMWDPs in the circulation is clearly not remarkable in terms of determining urinary excretion of LMWDPs.

The concept of LMWDPs derived from circulating albumin arising from extra renal degradation then becomes untenable considering the following facts. i) If LMWDPs are present in plasma they are readily observed (Table 2; Fig 1A) due to their prolonged elimination rate. For all our studies on circulating albumin we have never observed them as demonstrated in this study and elsewhere [5,6] even when 30-times the level of radiolabeled intact albumin was analyzed as compared to that used by Weyer et al [18]. ii) When considering albumin catabolism determined by the elimination of radiolabeled albumin from the blood circulation in rats, it occurs at a rate of 290–430 mg/day for a 300 g rat [44]. The appearance of albumin peptides in the urine ranges from 50–100 mg/day/300g rat [6,7]. If these urinary peptides were derived purely from extra-renal degradation where only 4% would appear in urine (coincidently we found only 0.3% (Table 2) of the injected dose of labeled albumin in urine at 2 h and 3.5% (Table 4) at 24 h), then the rate of extra renal albumin degradation would have to be 1200–2500 mg/day/300g; far in excess of the measured catabolic rate of circulating albumin. iii) Is there a partial contribution of extra renal degradation to urinary albumin fragment excretion? In this study our detection limit for low molecular weight degradation products from albumin is <0.025% of degradation products arising from the circulating material. The amount being filtered would be 4% of this which would be 0.001% of the degradation products arising from the circulating material. Therefore for a catabolic rate associated with the extra renal degradation of circulating albumin would lead to urinary excretion of ~1 mg/day/300g rat (30mg/ml (plasma concentration) x 2880ml/day (GFR) x 0.001%) which is far lower than the measured excretion rate. Even if our estimates of the amount of initial dose was in error by 3-fold it would still not contribute significantly to urinary excretion.

One of the most remarkable features of intact albuminuria in nephrotic states is that it can change by 2–4 orders of magnitude as compared to healthy controls [7,49]. It is apparent now that these large changes cannot be mimicked by equivalent changes in glomerular permselectivity involving charge and size [4,7,50,51,52]. The inhibition of specific renal degradation of filtered albumin does provide a plausible explanation of the large changes. An example of these changes occurring are shown in Fig 7 where albumin is primarily excreted as albumin fragments in healthy control rats and in PAN rats the albumin is excreted intact. The data in Table 4 would suggest that if there was no inhibition of the degradation pathway in PAN then the peptides would account for at least 5% (5 x 100/84) of the radioactivity in Fig 7 which would be readily detected. The inhibition is reflected in another way. The increase in the excretion of albumin-related radioactivity in PAN is ~17-fold (Table 4) whereas the increase in the excretion of intact albumin ~1500-fold [4,6,20] as measured by immunoassays that do not detect fragments (even comparison of peak heights of intact albumin in Fig 7 demonstrate the large differences involved as well). This means that there is at least a ~80 (1500/18)—fold inhibition of the degradation process. Overload effects on the degradation pathway due to a potential change in glomerular permeability can be eliminated as i) even in overload proteinuria (when plasma albumin is artificially increased by intravenous injection of exogenous intact albumin), peptide excretion is readily observed [53] and ii) there is little or no change in glomerular permeability in PAN [6,20,54] in spite of glomerular structural changes [35]. PA appears to specifically affect the kidney [55] where the inhibition in degradation is more likely related to the lack of dynamic uptake of albumin by the proximal tubules [2]. This means that the change in integrity of albumin excreted in the urine in renal disease is the primary factor governing the relative increase in intact albuminuria [21,56]. Similarly it occurs in nephrotic states as studied in antiGBM disease [6,56] and *Cd2ap* knockout mice [23]. In all these conditions, there is no change in glomerular permeability.

In summary, we have examined the plasma clearance and filtration of a range of LMW proteinacious components including LMWPs, LMWDPs that will include peptides and amino acids and tryptic digests of albumin. The appearance of these materials in urine is low. The results of Weyer et al [18] appear to be due to the excessive degradation by the kidney of their radiolabeled albumin preparation. Overall, the LMWDPs found in urine are the result of specific kidney degradation of filtered albumin. Metabolic perturbations of this degradation pathway will be the primary mechanism of non-hypoalbuminemic associated albuminuria. This degradation pathway is inhibited in nephrotic states- and while it does not cause hypoalbuminemia it primarily accounts for the relative change in intact albuminuria in these albuminuric states.

## Supporting Information

S1 File The files in Supporting Information contain data and graphs for the following experiments: 3H RSA tryptic digest data.xls; 3HLyz in Control column runs; 3HLyz in Control column runs.zip; 3HLyz in PAN column runs; 3HLyz in PAN column runs.zip; 14C RSA Experiments.xls; 14C RSA in PAN Experiments.xls; Albumin column runs; Albumin column runs.zip; Cyt Experiments.xls; Inulin Plasma Data.xls; Lyz Experiments.xls. (ZIP)
